# Overwhelming number of refugees in Poland - which strategy is being pursued

**DOI:** 10.1192/j.eurpsy.2023.120

**Published:** 2023-07-19

**Authors:** J. Samochowiec

**Affiliations:** Department of Psychiatry, Pomeranian Medical University, Szczecin, Poland

## Abstract

**Abstract:**

By the decision of the Polish Government, all persons arriving in the territory of the Republic of Poland after the commencement of the Russian aggression against Ukraine have free access to public psychiatric assistance, including reimbursement of pharmacological treatment on the same terms as Polish citizens. According to the UHNR data over 4 million displaced people, refugees, came to Poland so far and some of them benefited from such help.

Displaced individuals suffer from the consequences of traumatic events, exhibit psychological problems or develop mental disorders, including post-traumatic stress disorder, depressive and anxiety disorders or relapses of psychotic episodes.

The lecture will present data from the Interdisciplinary War in Ukraine Research Laboratory by pointing out the most important problems faced by Ukrainian refugees in Poland, why they chose Poland when escaping the war, how they evaluate Polish help, what their integration in Poland looks like. The survey also measured the level of war trauma (RHS-15). The results of the analysis show that most refugees present post-traumatic stress disorder symptoms (76 %), while only 0.15% of this population was treated in public psychiatric facilities.

Mental healthcare services are suddenly faced with major challenges and need to develop or expand strategies to address them. The Polish Ministry of Health prepared and started implementation of a reform of the mental health care system for adult as well as children and adolescent psychiatry. This fundamental reform should comprise three main actions, i.e. integration of mental health services into primary healthcare; establishment of community psychiatric services together with the provision of inpatient services in general hospitals; and limitation of the role of mental hospitals to specific tasks only (long-term or specialist treatment).

**Disclosure of Interest:**

None Declared

